# A Principal Investigator as a PrEP-Using Peer Change Agent for HIV Prevention among Black Gay and Bisexual Men: An Autoethnography

**DOI:** 10.3390/ijerph20075309

**Published:** 2023-03-29

**Authors:** Derek T. Dangerfield

**Affiliations:** 1 Department of Prevention and Community Health, Milken Institute School of Public Health, George Washington University, Washington, DC 20052, USA; ddanger2@gwu.edu; Tel.: +1-667-355-5188; 2 Us Helping Us, People Into Living, Inc., Washington, DC 20010, USA

**Keywords:** stigma, social networks, intervention, adherence, culture

## Abstract

HIV pre-exposure prophylaxis (PrEP) use remains suboptimal among Black gay and bisexual men (GBM). Multilevel factors such as medication costs, intersectional stigma, patient-clinician communication, medical mistrust, side effect concerns, and low perceived HIV risk (PHR) are well-established PrEP initiation barriers for this group. Peer change agents (PCAs) are culturally congruent interventionists who can circumvent multilevel PrEP barriers among Black GBM. I led an intervention as a PrEP-using PCA to improve PHR and PrEP willingness among 69 Black GBM from 2019–2022 and conducted an autoethnography to better understand multilevel barriers and identify the personal/professional challenges of being an in-group HIV interventionist serving Black SMM. Findings provide novel perspectives regarding PrEP barriers, the role of cultural homophily in behavior change interventions, and how interpersonal dynamics can impact staff fatigue, protocol fidelity, and research participation. Recommendations to prepare and support culturally congruent research staff are also provided.

## 1. Introduction

Pre-exposure prophylaxis (PrEP) is highly effective for HIV prevention [1,2,3]. In 2012, the U.S. Food and Drug Administration (FDA) approved Truvada^®^ for HIV PrEP [4] then approved a smaller pill with fewer side effects called Descovy^®^ in 2019 [5]. Despite increased awareness and access since 2012, PrEP use remains suboptimal among Black gay and bisexual men (GBM) [6,7]. Multilevel factors such as medication costs, intersectional stigma, patient-clinician communication, medical mistrust, side effect concerns, and low perceived HIV risk (PHR) are well-established PrEP use barriers for Black GBM [7,8,9,10,11]. For example, some Black GBM do not view themselves as PrEP candidates because they perceive their current sexual behaviors as lower risk than their past or their peers’ behaviors [8,9,12,13,14]. Some are also reluctant to accept a clinician’s PrEP recommendation due to experiencing poor clinical treatment [10,11,15]. Additionally, Black GBM consistently report reluctance to use PrEP partly because they believe that researchers and clinicians do not understand their experiences [10,15,16]. Approaches that address these barriers are urgently needed to improve PrEP use among Black GBM, particularly those under the age of 35 [17,18].

Some interventions leverage in-group members as peer change agents (PCAs) to change behavior [19,20,21]. For HIV prevention, PCAs are culturally congruent, trained professionals who understand the target population’s experiences, disseminate health information within the community, and promote healthy behaviors [19,20,21,22]. PCAs have helped improve behavioral health, HIV testing, treatment adherence, and PrEP initiation among several hard-to-engage populations [19,20,21,22,23]. PCAs are crucial for successful interventions among Black GBM because they are considered a more trusted resource than non-Black clinicians to obtain health information, discuss risks, and help them understand why PrEP could be helpful [9,15,24]. For example, PCAs build trust and circumvent barriers by using culturally acceptable and tailored language to describe clinical and research protocols, treatment recommendations, and shared experiences [9,15,19]. Studies show that Black GBM’s preferences for PCA characteristics include aesthetics, professionalism, language and social familiarity, PrEP use, and being a “future self” with whom they can relate [9,14,15].

Focus on the PCA is important in HIV prevention science for several reasons. For example, the experiences of a PCA can provide deeper insights into the multilevel barriers to behavior change among marginalized, priority target groups such as Black GBM. Additionally, PCAs can bridge knowledge and communication gaps between researchers, clinicians, and Black GBM using their dual experiences as clients and professionals. PCAs can also reveal the professional challenges associated with relying on culturally congruent members that could impact PrEP research participation and intervention effects among Black GBM. However, HIV prevention scientists have not adequately leveraged the perspectives of PCAs to optimize their utility and improve PrEP use among Black GBM.

### Autoethnography

Autoethnography is a research and analytic process that describes and explores the scientist’s experiences to systematically study and analyze a cultural phenomenon [25,26]. The researcher can obtain a deeper understanding of cultural norms, values, resources, emotions, and issues along with their self-identity by intentionally studying, recalling, reflecting, and writing about those experiences [27]. Autoethnography can provide a broader and nuanced understanding of cultural experiences in ways that cannot be captured in alternative qualitative approaches such as in-depth interviews or focus groups [28]. Specifically, autoethnography can provide details that may be missed because participants may consider some experiences as ordinary or unremarkable and not discuss them [25,26,29,30]. Some social scientists have published autoethnographies of health issues such as personal cancer journeys to better understand patient and professional perspectives [31,32]. Autoethnography in HIV prevention research can provide a broader and nuanced understanding of the multilevel experiences that create challenges improving PrEP among Black GBM. Autoethnography can also provide HIV researchers with greater insight regarding personal identity relative to health issues and minority populations.

The purpose of this study is to obtain a deeper understanding of the multilevel barriers of PrEP use among Black GBM and identify the professional issues for a PCA. Autoethnography can clarify the multilevel experiences of Black GBM and identify strategies to better engage them by comparing and contrasting personal experience relative to existing research [27,31,33]. Although autoethnographic experiences have been published for HIV treatment [34,35] and prevention [36], no autoethnography targeting PCA experiences in interventions serving Black GBM has been documented. Moreover, few health professionals are culturally congruent with Black GBM [37,38] and others might have different experiences that could prevent maximum exposure to the multilevel challenges prevalent among this marginalized minority population. Findings will provide novel perspectives regarding PrEP barriers, the role of cultural homophily in behavior change interventions, and how interpersonal dynamics can impact staff fatigue, protocol fidelity, and research participation.

## 2. Materials and Methods

### 2.1. Theoretical Framework

I led a team that refined and implemented POSSIBLE, a multicomponent intervention to improve PHR and PrEP initiation among Black GBM in Baltimore, MD [9] guided by Life Course Theory (LCT) [39,40,41], the Health Belief Model (HBM) [42], and Possible Selves Theory [43]. LCT suggests that timing, major life events, and age-related exposures to risk impact health behaviors and outcomes [41,42,43,44,45]. The HBM posits that perceived disease susceptibility can increase health behaviors [46]. Possible Selves Theory represents individuals’ ideas of what they could or want to become and can motivate behavior change [43,47]. Taken together, this framework informed our hypothesis that PrEP initiation could be improved by providing Black GBM with a smartphone app called PrEPme^®^ to self-monitor sexual behaviors and a PrEP-using PCA to review their risks and help change their PHR [9].

Formative research combined with extant literature suggested that the PCA should be a professional yet relatable PrEP-using Black GBM with similar multilevel experiences and a “future self” to whom participants could aspire [9,14,15]. Considering that HIV infections are highest and PrEP initiation is lowest among Black GBM under age 35 [17,48], we posited that the PCA should also be young with familiar experiences navigating relationships, HIV prevention behaviors, and PHR. Therefore, POSSIBLE was designed to cue Black GBM’s willingness to use PrEP by self-monitoring their behaviors and reviewing them with a young, PrEP-using “future self” who could tailor messaging relative to their sexual risks and PHR. This autoethnography was inherently guided by the tenets of LCT, the HBM, and Possible Selves Theory given the intervention design, my PrEP adherence, and dual role as a principal investigator and PCA along my own life course as a Black gay man from Baltimore City.

### 2.2. Study Participants & Setting

I served as the PCA for 69 HIV-negative Black GBM ages 18–67 who participated in POSSIBLE between 2019–2022. Participants were recruited using a combination of active and passive strategies [9,49,50] and were eligible based upon the following self-reported criteria: Black or African American race; male sex at birth; ≥18 years old; HIV-negative; being same-sex attracted to a man; and willing to use PrEPme^®^ during the study. Baltimore City was selected as the study site given its high priority for the Ending the HIV Epidemic Plan for 2030 [51] and geographic convenience for the research team. However, the quarantine of the COVID-19 pandemic in 2020 forced research protocols to become virtual and study visits were conducted via Zoom [52].

### 2.3. Intervention Protocol

Intervention study visits included 2 virtual sessions via Zoom one month apart. At baseline, I conducted a scripted motivational interview–consistent conversation to assess everyone’s lifestyles, goals and values, HIV risk behaviors, PHR, and PrEP interest [9]. At the end of baseline, I asked everyone to download PrEPme^®^ to document their sexual risks in the app-based diary each week. In the second session, I reviewed their diaries with them virtually and led another scripted motivational interview–consistent conversation to explore motivations for HIV-related risk behaviors, identify how their behaviors aligned with their goals and values from baseline, and reassess their PrEP interest. I discussed their relative and acute risks for HIV, answered questions regarding PrEP efficacy and side effects, and tailored prevention messaging to help increase PHR [53] and willingness to be referred to PrEP services in both sessions. At the end of each session, I helped interested participants obtain PrEP services at locations of their choice. I occasionally disclosed my PrEP use, discussed personal perspectives regarding HIV prevention, and dispelled PrEP misinformation depending upon participant requests or comments.

### 2.4. Autoethnographic Data Collection

Autoethnographic data collection included maintaining mental notes, jottings, and journal entries of PrEP care clinical visits and PrEP use along with POSSIBLE study visit summaries that were stored in participant folders [25,54]. Written and mental notes were documented after each study visit with participants, before and after PrEP visits, and after research team meetings in a designated research journal. Guided by the theoretical framework, formative research, and extant literature, note-taking focused on the social and cultural aspects of my PrEP use including barriers and facilitators along with interactions with participants as the principal investigator and PCA. Specifically, PrEP-related notes focused on known barriers such as stigma, costs, side effects, and PHR. Notes also focused on the range of feelings and attitudes towards the conversations and interpersonal dynamics between participants and I, including personal disclosures and professional concerns. Of note, I also attended weekly therapy sessions to support my mental health along my personal and professional journey.

### 2.5. Data Analysis

Data analysis involved reviewing, organizing, and coding mental notes, jottings, and study visit summaries relative to the theoretical frameworks and extant literature regarding PrEP initiation barriers. Specific experiences of PrEP barriers were highlighted, coded, and organized by hand using a pawing technique then prioritized for description [55]. Attitudes towards my role as the PCA were also coded, organized, and reviewed for salience. I grouped all such instances together from relative documents (i.e., journal entries, case report forms, jottings) and examined them for similarities and differences by each domain [54]. To optimize data collection, analysis, and the trustworthiness of the interpretation of this autoethnography, I also included a Black female qualitative research consultant with expertise in patient–clinician communication, experience studying sexual minority populations, and personal knowledge of the Baltimore City context. We met weekly to discuss multilevel experiences of my PrEP use, process feelings regarding working with Black GBM as an in-group researcher, and explore attitudes towards the research [50,56]. To ensure the accuracy and quality of data in recounting certain events and experiences, I shared my experiences, feelings, and notes with the consultant who affirmed some notes and challenged me to better describe more accurate versions of some of the stories [28,57]. We also discussed limitations and potential biases because my understanding of clinical protocols along with experiences of stigma, racism, and discrimination impacted my relationship to the research and participants. The iterative reflexive process allowed me to identify and address biases, mature my perspective regarding the study factors, and obtain high-quality autoethnographic data [50,56].

### 2.6. Researcher Life Course, Health Belief, and Possible Self

The multilevel and historical context of HIV epidemiology among Black GBM has impacted health beliefs, personal actions, and professional activities along my own life course and positioned me to uniquely qualify for this autoethnography. Specifically, my younger brother and I were raised by a single mother (before she remarried in my teens) in 1990s Baltimore City where I continuously experienced homophobic messages from family, church, schoolmates, and community members throughout my childhood and adolescence. I also experienced abuse from family and schoolmates who assumed that I was gay before I identified that way. I observed how community violence and stigma combined with limited social support for sexual minorities led to low healthcare utilization, sexual risk-taking, substance use, HIV/STIs, and suicide among my high school peers. Given the combination of homophobia, violence, and poor outcomes in Baltimore City I believed that Black and GBM (including myself) were worthless and unsafe. The only safety mechanism I perceived then involved academic achievements. Therefore, I focused academically as an unconscious coping strategy and defense mechanism from community violence, stigma, and rejection. I viewed prestigious universities as escape routes that could provide safety and stability as I ran away from bullies and other adverse experiences. Had it not been for my academic interests and opportunities, I could have experienced more of the negative outcomes prevalent among Black and gay/bisexual men in Baltimore.

I attended Georgetown University in Washington, D.C. where I studied finance as an undergraduate because I thought the school and major would give me the safety and security I sought as a child. For extra money during my sophomore year summer vacation, I was a research assistant at the Johns Hopkins School of Medicine where I recruited and interviewed Black patients in the Baltimore City STD Clinic. I studied how some Black men and women in Baltimore City experienced similar and worse multilevel challenges as I did that led to negative sexual health outcomes. I became vigilant to not allow those experiences to catch up with me in the form of HIV or another sexually transmitted infection as an adult. For example, I did not have sex for almost two years after I learned that someone I dated lied to me about his HIV-positive status. HIV became another bully I ran from because of the stigma associated with being a “promiscuous” or “dirty” Black gay man. I never seroconverted. However, I acquired a paralyzing anxiety of losing the long race against the ravenous retrovirus that rages through my community. I ultimately changed my major from finance to sociology and continued conducting research globally to prepare for an academic career and a life of safety and security. I began dating again as a senior in college but never fully trusted anyone (including a new boyfriend of two years) because I was hypervigilant about HIV prevention and unconsciously disassociated myself from Black GBM. Although I practiced safer sex consistently, including abstinence, HIV risk haunted me and tarnished my dating experiences throughout my entire 20s.

I moved to Los Angeles, CA in 2013 at the age of 22 to pursue my PhD at the University of Southern California and to give my relationship a fair chance after finishing a year-long Fulbright research fellowship in Southeast Asia. Los Angeles exposed me to a broader range of lifestyles and wellness activities that allowed me to socialize with the LGBT community with less judgement than I experienced as an adolescent in Baltimore. However, my apprehension of identifying with Black GBM prevented me from fully connecting with others in the city (including my boyfriend) and ultimately led to the end of my relationship. As a newly single graduate student, I was simultaneously establishing a new group of Black gay friends, dating Black gay men, and studying HIV risk behaviors among Black GBM for my dissertation research. My combined social, romantic, and academic experiences exacerbated my exposure to Black GBM who were newly diagnosed with HIV regardless of their PHR and constantly triggered my fears of seroconverting. Despite consistent condom use and personally testing my partners for HIV in my apartment, I thought that seroconverting was inevitable unless I used the newly available PrEP for prevention. I initiated Truvada^®^ as PrEP in 2015 for 6 months after a year of therapy to recover from my breakup, overcome internalized homophobia, and develop a group of Black gay friends for the first time.

I completed my PhD with distinction in 2017 at the age of 26 and pursued a postdoctoral fellowship at the Johns Hopkins School of Nursing to test my recovery and hopefully help other Black GBM back home. Since my doctoral training, I have personally conducted ~200 in-depth qualitative interviews, 50 focus groups, and >400 h of in-person clinical and community-based observations among Black GBM throughout the U.S. I have learned that the challenges I experienced as an adolescent are still salient and that novel approaches to help Black GBM are needed to fully free myself and others from multilevel barriers. I shifted my research and training to focus on designing and implementing HIV prevention clinical trials among Black GBM. Therefore, I designed and implemented POSSIBLE as a 28-year-old Black gay man from Baltimore City who was an Assistant Professor at Johns Hopkins School of Nursing

### 2.7. PrEP Initiation & Adherence

I reinitiated PrEP (Descovy^®^) at the end of 2019 in response to formative research and my team suggesting that I serve the intervention as the PCA [9] rather than using the project as a vehicle for professional and personal safety as I had done with research as a graduate student. I maintained daily adherence for approximately a year and a half until I ended a romantic relationship at the beginning of 2021. I had enough pills left over to use PrEP on-demand (2 doses the day before sex, 1 dose the day of, and 1 the day after [2,58]) if I anticipated having a casual partner (which did not happen during that season). PrEP became an intimate part of an emotional, psychological, and sexual defense mechanism that I was uncomfortable relying on given the salient experiences of stigma as a Black gay man and constant reminders of being a part of a highly vulnerable and marginalized group.

## 3. Results

Data presented describe the autoethnography of my role as a PrEP-using principal investigator and PCA in POSSIBLE by multilevel factors and salient professional challenges. Quotes from participants are provided to corroborate my personal assessments and make the text evocative by “showing” thoughts, emotions, and actions [26,54]. Intervention effects will be described in a separate publication.

### 3.1. “High Risk Homosexual Behavior”: Navigating Stigma from Clinicians

I reinitiated PrEP at a primary care facility that was associated with a university hospital in Baltimore City. Even though I knew the HIV/STI screening guidelines, I always had an anxiety and fear of judgement as I anticipated the questions regarding my relationship status, partners’ gender, and sexual positioning practices (if clinicians asked at all). Clinical experiences were typically uncomfortable regardless of clinician demographic characteristics. The first provider I saw in Baltimore was a middle-aged white man who was unfamiliar with the PrEP treatment guidelines. I explained the guidelines to him to save both of us time, but he reviewed the Centers for Disease Control and Prevention website for confirmation before he agreed to fill the prescription. I was unsure if he was doing his due diligence as a clinician or if he did not believe me because I am Black. I was too uncomfortable with needing the prescription to ask. Although I did not have most of the PrEP indications (i.e., STI history, drug use, condomless sex), he said, “*Given that HIV is so high in your group, I think it’s a good idea. I don’t want you to get it*”. By his tone, I was also unsure if he really cared about my health or if he was just reviewing my profile like I was a number. It was always difficult to decide if and when I should relax as a patient or assert myself as a nursing professor to protect myself from the stigma [59]. I always felt a power imbalance with clinicians and staff because I needed health care from them that did not allow me to fully express my discomfort with the experiences or ask for the treatment that I really wanted. Communicating with clinicians about sexuality and PrEP became another gut punch from the HIV bully and triggered feelings of shame about being a member of a “high-risk” population.

Some clinicians assumed that my partners were female and I had to be brave enough to rescind the small amount of “passing” privilege that I had and tell the truth about my sexuality. During a follow-up visit at a different primary care clinic where my provider and nurses were Black, I overheard the team using my name as they discussed my case in the hallway, including the extragenital STI testing I needed as part of routine PrEP care. I was unsure if others heard their conversation. The manner of the conversation felt inappropriate, unprofessional, and hurtful despite the fact that they did not say anything particularly derogatory. Every visit I wondered if the care team made derogatory comments about my extragenital screening after my visits. I made conscious efforts to overcome feelings of shame as a Black gay man and disregard clinical care teams’ inabilities to make me feel comfortable even though I had low acute risks.

After-visit summary reports for my PrEP visits always indicated “high risk homosexual behavior” in the reasons for the visit at the top of the page regardless of what I reported. The documentation triggered internalized homonegativity and intersectional stigma for being part of a “high risk” population and resentment towards clinicians who documented my case in an offensive way. I tried to alleviate the stigma and justify their language choice by rationalizing (or assuming) that the billing codes required use of this terminology even though that was never explained. I delayed follow-up care visits to avoid navigating interpersonal challenges with clinic staff, possibly hearing the team discuss my case, and needing to emotionally recover from hurtful, stressful, and stigmatizing clinical experiences. I misled clinicians twice during months I was not sexually active to avoid being asked personal questions about homosexuality because I knew the clinical screening guidelines enough to get the prescription. However, after-visit summaries still indicated “high risk homosexual behavior” as the reason for the visit and reminded me of the resentment I had towards being a Black gay man. As the PCA, I still convinced men to suppress their discomfort with clinicians despite my knowledge of what they could experience and encouraged them to obtain a medication for a condition they did not have.

Participant (Age 28): I’m not really trying to do it [PrEP] because I don’t feel like dealing with them people in the clinic.

Me: I totally get it. It’s awful. It’s something we just gotta deal with to keep ourselves safe.

Participants: Yeah I hear you.

### 3.2. Costs

I never paid out-of-pocket for PrEP medications. As a graduate student, I had healthcare coverage, and the Los Angeles Gay and Lesbian Center still introduced me to a co-pay assistance program that allowed me to obtain Truvada^®^ for free. I do not recall paying for associated lab fees (my school stipend was low enough for me to remember an expense for a condition I did not have). I remembered the co-pay assistance program when it was time to initiate POSSIBLE and I re-enrolled because I did not want to pay the full expense for a medication I did not think I needed. My quarterly lab fees for clinic visits and HIV/STI testing were between USD 50 and 100 depending on how many tests I received. Although I was making a comfortable salary as an assistant professor, I considered the medication and lab fees expensive because it was an unnecessary expense. I might not have consistently refilled the prescription if I was not the PCA because the variable expenses for the lab fees did not feel worthwhile given my PHR and negative feelings. My knowledge of the medication costs, laboratory fees, and co-pay assistance programs allowed me to discuss some of men’s concerns regarding PrEP care expenses, especially with those with qualitatively low PHR.

Participant (Age 30): I don’t really want to pay for it because I’m not out here like that.

Me: Totally get it. There’s a copay assistance program you may be eligible for so you don’t have to pay for the medication.

Participant: Oh, I didn’t know that. I might consider it then.

I underestimated the role of insurance coverage on PrEP willingness and use in my work with participants. Almost all uninsured participants were apprehensive or unable to initiate PrEP due to insufficient coverage and did not want to navigate the challenges of obtaining financial assistance for the sole purpose of initiating PrEP. Individuals who were interested in PrEP experienced several challenges regarding continuous income verification and other administrative forms that decreased their willingness to follow up with their appointments. Some blamed me for increasing their exposure to social challenges that they would not have experienced had I not encouraged them to use PrEP and exacerbated their frustrations with navigating U.S. social services. I also blamed myself because I initially underestimated the barriers, then I ignored them to support my professional and scientific goals.

### 3.3. Medication Side Effects

Both times that I initiated PrEP I had one week of diarrhea and abdominal pain but no other noticeable side effects afterwards. I considered these relatively mild side effects considering the alternative of living with HIV and having to take similar medications daily to live. I also maintained pride in understanding that PrEP was always a choice and that I did not have to take the medicine if I really did not want to. I used this thinking to help persuade ambivalent participants to take PrEP.

Participant (Age 28): I don’t think I need it and I don’t want medicine in my body.

Me: I totally understand that. I’ve thought about that too. But the way I look at it for me is you can take this medicine to avoid HIV and stop whenever you want or you get HIV and you have to take the medicine every day for the rest of your life. You will also work with your clinician to make sure nothing bad is happening to your body while you take it.

Participant: Yeah, you right. I’ll try it.

I was uncomfortable with the idea of convincing participants to disregard their concerns about taking PrEP to prevent them from needing to adhere to treatment medications if they seroconverted. I was also ashamed to encourage Black patients to use a medication that could provide a range of side effects that they did not necessarily want in order to prevent a disease they did not have. Internal conflicts regarding honoring participants’ disinterest in PrEP versus professional goals to increase uptake for HIV prevention persisted for me. I also struggled to manage discussions regarding side effects with Black GBM whose health histories I was unfamiliar with or unqualified to discuss as someone who was not a clinician.

### 3.4. Relationship Status & PHR

I perceive my HIV risk as high regardless of sexual activity (or the lack thereof). However, I did not perceive PrEP use as necessary in 2019 because I was in a committed, monogamous relationship and I gave him an at-home HIV test myself to confirm his status before we started having sex. Quarantine in 2020 during the COVID-19 pandemic also made it difficult to socialize and helped assuage my concerns about partner infidelity. Still, using PrEP during that relationship provided a sense of relief knowing (or at least believing) that I was somewhat protected from HIV despite persistent fears of seroconverting. PrEP use allowed me to participate in a relationship with a new partner and at least try to remove the worry about HIV that prevented me from connecting with him and others previously. On some level, I felt that I did not have to trust my partner because at least I would not get HIV if he cheated or lied about his status later. Navigating high and low levels of PHR relative to the perceived need for PrEP during a relationship was something that I became very familiar with as I talked to participants who were partnered. Occasionally I shared my relationship status and thoughts regarding PrEP use in the context of a partnership.

Participant (Age 26): I don’t think I need PrEP because I’m in a relationship.

Me: Are y’all using condoms?

Participant: No. We are monogamous though.

Me: I totally get it. That’s something that I struggle with myself. I saw some research from Atlanta a while ago suggesting that most people who were newly infected with HIV acquired it from people they considered “main partners.” I have my thoughts about what it means to be a main partner, but as Black gay men we really can’t afford to take any chances with our health. Since you said you don’t want HIV, I think you should still use it because we just never know what someone else is doing and I care about your health. What do you think?

Participant: Wow. I think you’re right. You really never know.

I struggled with encouraging men who were in monogamous relationships to use PrEP because I did not want to perpetuate feelings of distrust towards other Black GBM. I knew that my history of a partner lying about his status was impacting my distrust of partners. I also understood the difficulties for Black GBM to overcome fear and internalized homonegativity and fall in love and I did not want to be another person telling them to limit themselves. I was envious of individuals who explicitly mentioned that they trusted their partners or did not want to use PrEP because they wanted to trust their partners. I was simultaneously uncomfortable with the idea of not warning my community of the very real epidemiological consequences of condomless sex, so I encouraged everyone to use PrEP as part of my professional and scientific goals.

I discontinued PrEP after my relationship ended because I had no intentions of dating for the rest of the year. I also thought I had enough experience to share with participants even though some had questions regarding side effects from using PrEP across several consecutive years. Being newly single changed the way I viewed my research and my role as the PCA. I felt more vulnerable to the multilevel experiences of being a single, Black gay man again. As my interest in dating again grew, I started to feel the vulnerability of wanting to connect with someone that triggered thoughts about PrEP. I remembered how PrEP allowed me to participate socially, but I did not want to use it as a crutch to have casual partners. I needed help navigating being a single, Black gay man, but did not want it from PrEP. That pill was a daily reminder that I was vulnerable to the desire for a new relationship that I could not see coming soon. I also did not want to navigate the stigmatizing clinical experiences of PrEP care again or pay for a medication for a condition I did not have. Moreover, I did not want for-profit pharmaceutical companies to benefit from my intersectional vulnerabilities as a minority individual. Unconsciously, I think I looked for guidance through the conversations with my participants who were willing to date and fall in love with or without PrEP.

Being single changed my relationship to the intervention because I felt the need to intentionally guard myself from the vulnerabilities of wanting a new relationship like participants and peers and navigate my professionalism in a way I deemed unnecessary when I was partnered. Being single also forced me to navigate conversations with participants differently, particularly older men or peers in my age group (i.e., 25–30). I was cautious about disclosing too much about myself because I wanted participants to view me as professional as possible even though I could relate to their experiences. Some asked me if I was single and interested in meeting outside of the study (socially and romantically), which felt inappropriate and uncomfortable even though sometimes I wanted to. Those requests raised ethical concerns regarding the appropriateness of socializing with participants even though we could have met outside of the study given our cultural congruence and small social networks. Moreover, I was unsure if participants were interested in me as the professor/principal investigator or felt that I was more approachable as the PCA, which made me feel more ordinary than I wanted to be perceived. I also did not want to discuss my own desires for partnership during a professional experience, even though I disclosed other personal aspects about myself to help improve PHR and PrEP willingness during the intervention. I navigated conversations regarding the acute risks for HIV among single participants and tried to mentally block myself from the vulnerability of knowing that I was in a similar circumstance of loneliness and desire for a relationship regardless of behavior.

### 3.5. Anticipated Stigma from Partners

I anticipated stigma from partners because PrEP was proof that I was distrustful and afraid to connect. My boyfriend at the time never directly asked me about my health history or PrEP adherence. At the beginning of the relationship, I told him I was using PrEP, feeling that I was “warning” him or trying to get ahead of perceptions that I was promiscuous. I explained that using PrEP was a part of my research as a defense mechanism to manage any negative perceptions about my risk or history. I prepared to manage a conversation about whether I wanted an open relationship or if I trusted him to be monogamous, but neither came up. I believe that I did not have to continue justifying my PrEP use partly because it was part of my job. I also think I demonstrated my commitment to health by giving him an HIV test. I occasionally thought that if I was not involved with this research I may not have used PrEP as consistently to demonstrate more vulnerability and trust in the relationship. However, I never allowed anticipated stigma from any partner to decrease my adherence because I was more afraid of HIV acquisition than losing a partner. Some participants were ambivalent about using PrEP because they anticipated navigating a similar experience with their partners.

Participant (Age 24): I don’t want him to think I’m a whore.

Me: I totally get it. But I never worry about what someone else thinks of how I protect myself. I care about your health, and we don’t want HIV. So is that really enough to not do it?

Participant: I get it. I’ll think about it.

The anticipated stigma from partners contributed to some participants’ qualitative underestimation of their PHR or acute HIV risks. My understanding of the perceived or anticipated stigma from partners helped me manage participants’ concerns among those who were ambivalent because they wanted to demonstrate trust in their relationships.

### 3.6. Internalized Stigma

PrEP adherence was a daily reminder of my membership in a marginalized subpopulation that was being medicated for who we were. As a professional, I understood that Black GBM are among the highest priority populations for HIV prevention. As a patient, I struggled with my professional knowledge that I was using medicine that was targeted for a “high risk” population because of natural feelings and behaviors that unfortunately had disproportionate epidemiological consequences. As a responsible, professional adult I did not meet any of the indications for PrEP, yet my identity and desire for companionship as a Black gay man increased my HIV risk. None of my heterosexual friends ever considered using PrEP, even if they had STI histories, and clinicians never targeted them to use it. PrEP added another layer of feeling marginalized, mistreated, and misunderstood for being a Black gay man that I was still trying to grapple with as I approached my 30th birthday. Using PrEP also reinforced feelings that my desire for a romantic/sexual relationship was unsafe. It constantly reminded me that I was still at risk for a chronic condition because of a desire for a relationship and that I still might not find a safe one.

I also internalized shame and resentment during my relationship because I was using PrEP and my boyfriend was not. I did not want to pressure him into using PrEP because I did not want him to think I was distrustful of him or add to his challenges given that he lost his healthcare coverage after losing his job during the COVID-19 quarantine. Being the only one in the relationship using PrEP increased shame and resentment for using a medication that I thought was targeted for risky and low-resourced men, a self-perception I consistently fought. I understood why participants did not want to trigger similar feelings of shame and stigma in themselves. Every day I took my medicine I suppressed internalized shame about being a Black gay man who “needed” PrEP despite not meeting most of the clinical indications. I struggled to balance the fear of HIV vulnerability with the empowerment of protecting myself. I had a buffer of Black gay friends who either used PrEP at the same time I did or supported my PrEP use, which helped mitigate my feelings of shame and stigma.

### 3.7. Vulnerability & Desire

During and after my interviews, I also struggled with being reminded that I was part of a community of ordinary and extraordinary men who were vulnerable on many levels. Most of us had desires for relationships that felt elusive, all of us had disappointing clinical, social, and romantic experiences, and all of us were at risk for HIV. I shared many of the experiences and concerns that we discussed but did not want to see myself as someone who was still experiencing multilevel barriers despite some access to power, privilege, and prestige as a sexual health professor. I did not want to identify with those challenges because I worked tirelessly to escape them (or so I thought) and did not want to remind myself that as a Black gay man I could never escape the adversity or risks that I studied despite perceptions that academics live in “ivory towers.” Being the principal investigator allowed me to have a false sense of elitism and protection from the challenges of being a Black gay man and the vulnerability of being a PCA with similar desires, hopes, and fears as research participants.

## 4. Discussion

PrEP care and daily adherence reinforced an awareness of the salient multilevel challenges of Black GBM that triggered feelings of isolation, shame, stigma, internalized homonegativity, resentment, and treatment refusal. PrEP use also triggered medical mistrust and treatment hesitancy due to feeling medicated for being a member of multiple minority groups. Some GBM suggest that PrEP liberates them to participate safely and freely without fear of HIV [60]. Similarly, I found that PrEP reduced some concerns regarding HIV acquisition. However, the perceived benefits of using PrEP to prevent HIV did not outweigh persistent feelings of loneliness, resentment, stigma, and shame along with the challenges navigating barriers. Navigating multilevel PrEP barriers was ongoing and challenging with limited support. I needed to rely upon my identity as a health professor to help reduce inequitable feelings of shame, stigma, distress, and costliness as a PrEP-using PCA. Few aspects of the PrEP care process supported willingness, initiation, and adherence.

I was concerned that being the PCA would overshadow my role, identity, and professionalism as a principal investigator throughout this study. My role as the principal investigator provided a false sense of safety from the challenges of being a Black GBM that impacted PrEP attitudes. Being the PCA removed some of my perceived professional superiority and distance that principal investigators typically have over staff and participants during data collection. This autoethnography improved my humanity and self-image by forcing me to reframe my perception of Black GBM from being risky and vulnerable to viewing Black GBM as people and peers. I shared similar beliefs, interests, desires, and adversities; other’s experiences helped me process my feelings regarding race, class, sexuality, love, and PrEP. I learned to embrace my connection to the experiences of Black GBM and intentionally use adversity to support community health.

Despite embracing an empowering community health agenda, this autoethnography reinforced the constant awareness of my multiple marginalized identities that persisted regardless of my self-efficacy or socio-economic status. W.E.B. Du Bois described a “twoness”, a double consciousness that many Black people experience as they navigate life as racial minorities in America. The double consciousness refers to viewing oneself in the eyes of white people, an imposed self-awareness with competing ideals and goals as Black and American [61]. For Black GBM, this concept is germane to the clinical setting as we view ourselves through the eyes of primarily white clinicians in a socioecological context designed by white heterosexuals in service of white people. As Americans, some of us have access to quality healthcare. However, as Black men, the vulnerability to poor treatment from PrEP-prescribing clinicians (or any other social system in America) is inescapable [62,63,64]. Black GBM who have less awareness of screening protocols and less capacity to navigate and interpersonal dynamics with clinicians experience even greater unresolved barriers that must be eliminated [10,59,63].

I suggest that Black GBM experience a “threeness”, an added identity as a sexual minority that could create a triple consciousness [65], exacerbate negative self-perceptions, and reduce health maintenance [59]. The threeness can refer to the competing ideals, goals, and worlds as Black, queer, and American [59,65]. While the twoness yields a double consciousness of identities due to Black race as Americans, the threeness exacerbates competing self-consciousness given an added sexual minority status. Specifically, identifying as gay or bisexual adds to the marginalization of Black GBM in the eyes of mostly white clinicians and social systems (including healthcare) that perpetuate stigma and isolation. Additionally, the challenges associated with navigating and developing romantic relationships are arguably unique for Black GBM given prevalent histories of trauma, isolation, and community HIV/STI risk, all of which can impact trust. This autoethnography highlighted my self-consciousness in the eyes of white clinicians but also of other Black and GBM individuals. I struggled to fully overcome multilevel PrEP/social barriers given my competing goals as a Black and gay and American professional man. I am uncertain if I ever can. Hopefully it is “possible”.

This study became a safe vehicle by which I could live in my hometown as an adult Black gay man vis-à-vis my relationship with the men in POSSIBLE despite interpersonal, intrapersonal, administrative, and professional challenges. My age at the time influenced my clinical, professional, and social experiences given the context of navigating as a 20-something year old and changed my relationship to PrEP, my research, peers, and participants. Being the PCA could have saved someone’s life despite my challenges and reservations to serving in the role. Autoethnographic data analysis and writing helped heal intrapersonal challenges [31]. Consulting other researchers and participating in mental health services was crucial during this process. Sharing familiar experiences with peer-participants was also healing, hopefully in a bi-directional way.

### Implications

PCAs require additional support from colleagues and supervisors to ensure their personal safety and professional identity. The emotional, financial, and psychological burden required from the role was not equitable. For example, navigating social and potentially romantic interactions with other Black GBM in this culturally congruent work is unique given the potential for same-sex attraction. The extreme homophily of the PCA in PrEP interventions among Black GBM could increase staff fatigue and turnover as well as affect recruitment, retention, and engagement depending upon the rapport with participants [50]. Research teams should ensure frequent (e.g., weekly) meetings with PCAs to assess their personal and professional wellbeing and consider having two staff members present in intervention meetings to increase support. Moreover, the salary for PCAs should be increased given the required experience, expertise, costs associated with PrEP care, and fees for recommended mental health services.

PrEP-prescribing clinicians and clinical support staff should be aware of the multilevel adversities that Black GBM experience prior to, during, and after clinical visits. Clinicians should ensure that they follow CDC guidance regarding open communication with patients and that they incorporate knowledge of multilevel challenges of Black GBM into their conversation to build rapport, alleviate stigma, and support PrEP adherence. For example, clinicians could proactively acknowledge and assuage patient concerns regarding sexuality discussions, PrEP and sexuality stigma, and extragenital screening protocols. Clinicians should also inform patients that PrEP use could trigger feelings of stigma and shame throughout the experience and affirm their decisions to demonstrate compassion and empathy. However, ICD-10 codes for clinical diagnoses and billing must be updated to more accurately describe the nature of clinical visits among GBM and reduce stigma. Trauma-informed care models integrate knowledge about patient histories into their policies, practices, and procedures regardless of the demographic composition of care teams and can improve treatment adherence [66,67]. 

Additionally, clinical care teams could be a source of emotional and psychological support during and in-between visits to facilitate initiation and adherence among Black GBM [15,24]. Productive and familial-like patient communication can reduce stigma and increase patient treatment adherence [68,69,70]. Clinicians could also use PrEP to alleviate sexuality- and medication-based stigma and reduce medical mistrust [9,15]. PrEP provides a unique opportunity for patients to request that health professionals who do not have a disease to use and disclose experiences using a medication they recommend [15]. Unlike other chronic conditions such as cancer or hypertension, where medication is used for a present disease, it is reasonable for apprehensive patients to ask HIV-negative researchers and clinicians to use PrEP to help reduce medical mistrust and demonstrate their commitment to community health with Black GBM. Having clinicians who disclose their own PrEP use could create an equitable atmosphere with patients that removes barriers of race, gender, and sexual orientation. Black-led clinical care teams are considered more trustworthy than white clinicians and could also help improve adherence for Black GBM [15,24]. 

This study is not without limitations. My professional expertise in sexual health coupled with previous PrEP experience provided support for my adherence that may not have occurred otherwise and impacted my participant interactions. I could not fully divorce myself from my role as principal investigator and professor, which impacted my PCA experience. I did not focus on alternative PrEP modalities such as on-demand PrEP or injectable Cabotegravir [2,58,71]. Also, it will be difficult if not impossible for other researchers (including myself) to replicate this study and experience similar findings given the age- and culturally- specific nature of this research.

Future research should test the relative impact of the PCA on PrEP initiation among Black GBM. Larger efficacy trials are needed to identify the efficacy and equitability of leveraging in-group members as PCAs given the emotional and psychological burden that will be placed on research staff. Interventions should also identify and utilize PCAs with culturally acceptable characteristics beyond race and sexuality congruence that might be useful to Black GBM. Future research should also consider replicating the premise of this study using the newly approved PrEP injection for HIV prevention that only requires treatment once every two months and therefore inherently reduces exposure to some multilevel barriers [72].

## 5. Conclusions

Negative psychosocial experiences are salient along the life course of Black GBM and illuminated by PrEP. The PrEP care experience also triggered unresolved emotional and psychological factors as a minority American that are not adequately addressed by PrEP care or research teams. The cumulative role of trauma, stigma, homonegativity, PHR, and fear impacted clinical engagement and PrEP use and should be reconceptualized for Black GBM. I hope this autoethnography underscores how interrelated and salient multilevel factors are for us and how they impact, health behaviors, goals, and values. However, this autoethnography provided some healing by systematically studying, describing, and sharing [31]. I have hope that researchers and clinicians can find humanity in this work.

## Data Availability

Not applicable.
